# Raspberry Pi-Based Sleep Posture Recognition System Using AIoT Technique

**DOI:** 10.3390/healthcare10030513

**Published:** 2022-03-11

**Authors:** Pei-Jarn Chen, Tian-Hao Hu, Ming-Shyan Wang

**Affiliations:** Department of Electrical Engineering, Southern Taiwan University of Science and Technology, Tainan City 71005, Taiwan; cpj@stust.edu.tw (P.-J.C.); ma820401@stust.edu.tw (T.-H.H.)

**Keywords:** sleep monitoring, internet of things (IoT), random forest classifier (RFC)

## Abstract

The relationship between sleep posture and sleep quality has been studied comprehensively. Over 70% of chronic diseases are highly correlated with sleep problems. However, sleep posture monitoring requires professional devices and trained nursing staff in a medical center. This paper proposes a contactless sleep-monitoring Internet of Things (IoT) system that is equipped with a Raspberry Pi 4 Model B; radio-frequency identification (RFID) tags are embedded in bed sheets as part of a low-cost and low-power microsystem. Random forest classification (RFC) is used to recognize sleep postures, which are then uploaded to the server database via Wi-Fi and displayed on a terminal. The experimental results obtained using RFC were compared to those obtained via the support vector machine (SVM) method and the multilayer perceptron (MLP) algorithm to validate the performance of the proposed system. The developed system can be also applied for sleep self-management at home and wireless sleep monitoring in medical wards.

## 1. Introduction

Most people spend one third of their time sleeping, and the quality of their sleep is closely related to their health. Sleep quality is affected by many sleep disorders, such as sleep apnea, insomnia, and snoring. According to [1], over 70% of chronic medical disorders (such as cancer, chronic renal failure, chronic obstructive pulmonary disease (COPD), fibromyalgia, gastroesophageal reflux disease, heart failure, HIV-related disease, nocturnal asthma, restrictive lung disease, etc.) are highly correlated with sleep problems. However, few people can determine their own sleep habits and sleep quality because accurate diagnosis requires sleep monitoring throughout the night. Conventional sleep-monitoring equipment can induce feelings of discomfort, for example, pressure sensors require a power supply such as batteries, and it is difficult ensure that the users not feel them when laying on top of them [2,3], and there is usually a three-axis accelerometer that strapped to the person’s chest [4]. Additionally, sleep monitoring systems that are equipped with cameras, electrocardiograms (ECGs), or respiratory belts [5] raise concerns about personal privacy. In addition, there is a higher risk of problems related pressure ulcers on some parts of human body, especially the portions over bony or cartilaginous areas, if the patient maintains the same sleeping position for a long time. In order to avoid pressure ulcers, nursing staff need to help patients turn over every two hours [6,7]. As a result, a system that can monitor sleep posture and record data can improve a patient’s sleep quality and reduce the work of nursing staff, hospital stay costs, and the enormous burden on healthcare systems [6,7].

In recent years, many kinds of hardware for sleep posture monitoring have been used. Pressure sensor pads were employed to monitor the pressure changes exerted by a body on a mattress [2,3]; noncontact electrocardiography (ECG) was adopted to monitor the user’s QRS waveforms and thus detect their sleeping position [8]; A total of sixteen long and narrow force-sensing resistor sensors were used to estimate the kurtosis and skewness for bed posture detection through Bayesian classification [9]; four hydraulic bed transducers were placed underneath the mattress to investigate different sleep postures [7]; pressure image data have been used to detect sleep posture and to identify different limbs [10]; depth cameras were previously used to capture three-dimensional images of a monitored person [11]; and a three-axis accelerometer was strapped to a person’s chest to monitor his/her sleeping position [4]. However, these hardware-based systems have an insufficient level of comfort, high cost, low data collection stability, and image noise as well as privacy problems. A radio frequency identification (RFID) tag-based sleep monitoring system may overcome those problems and provide a non-intrusive, convenient, and comfortable way to monitor sleep quality [5,12,13]. The NIGHTCare platform [5] provided a night suit that contains four wearable RFID tags at abdomen, back, and left and right hip, a mattress-embedded conventional dipole tag, and a bed with four tags positioned at each side. Another sleep monitoring system has 64 RFID tags embedded into a bed sheet in a matrix form and uses 400 groups of teaching data to train the system and 100 groups of testing data with different postures to realize sleeping posture recognition and body movement detection using a CNN algorithm [5]. Its average recognition rate is 86.284%. A TagSheet system [13] based on 30 × 18 RFID tags was proposed to monitor sleeping postures with a combination of hierarchical recognition and was able to obtain up to 96.7% accuracy. In addition, a novel non-invasive sleep monitor system that consists of a smart glove sensor, a mobile application, and a remote server has also been proposed [14]. This glove sensor acquires a three-axis accelerometer signal, a photoplethysmography (PPG), and a peripheral oxygen saturation signal from the index finger. After cloud computing, the remote server performs different sleep monitoring activity tasks.

Additionally, there are three popular classification algorithms for sleeping posture monitoring: the multilayer perceptron (MLP) algorithm [7,12,15], the support vector machine (SVM) algorithm [16,17,18,19], and random forest classification (RFC) [12,13,14,15,16,17,18,19,20,21,22]. An MLP, also known as a feedforward neural network, is an artificial neural network (ANN) with a forward structure. The main advantage of an MLP lies in its ability to quickly solve complex problems and to recognize linearly inseparable data. ANNs have been refined and applied in many fields, such as in robotics, medicine, the military, and the automotive industry.

SVM is a supervised machine-learning model that analyzes data for classification and regression analysis; based on statistical learning frameworks, it is among the most robust prediction methods. SVMs can be used to solve various real-world problems, such as text and hypertext categorization, images classification, satellite data classification, hand-written character recognition, and biological sciences.

The RFC method is an ensemble-based technique that can be used to perform classification tasks. RF method utilizes a multitude of classification trees randomly and then generates a final result. These trees are randomly created by extracting samples from different subsets of the same dataset, and different types of features are employed each time to create the trees. Consequently, the RFC model does not overfit the data during the decision tree process [20]. Once the trees have been constructed, classification may be conducted to search the results of each tree, and the results are assigned to the class that has been determined by the greatest number of trees.

Referring to [5,13] and considering factors of convenience, compactness, and cost, this paper proposes a stable and comfortable sleeping posture recognition system based on RFC and 5 × 5 RFID signals that provides stable data through their ability to penetrate bedclothes but not the human body; only signal strength information is collected to address privacy concerns. The system employs a Raspberry Pi 4 Model B as an artificial IoT (AIoT) for data processing, integration, and prediction. Advantages include its low cost, low power consumption, and sleeping posture prediction accuracy, which reaches the levels of the other aforementioned sensors.

Three contributions/advantages of this system are summarized as follows:

(1) The AIOT system with a Raspberry Pi microprocessor and nonintrusive passive RFID tags embedded in the bed sheets provides safe, comfortable, low-cost, and privacy-protected monitoring so that users can maintain their normal sleeping habits.

(2) This system can be applied in nursing homes and in hospitals to monitor a patient’s prone position and to deliver reminders, reducing the workload of the nursing staff. The system also provides a data collection function to allow users and doctors to view historical records online.

(3) The RFC requires only 25 RFID tags and approximately 4000 data points to achieve an accuracy of 88.9%. The results demonstrate that RFID has great potential as a monitoring sleep posture method.

In this paper, the system description and methods are discussed in Section 2. The experimental results are presented in Section 3. Discussions are provided in Section 4. Finally, conclusions are drawn in Section 5.

## 2. System Description and Methods

**Figure 1:**
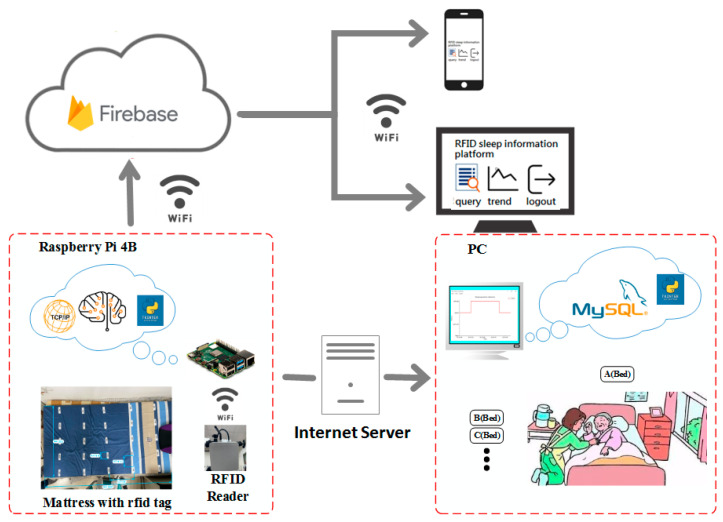
System architecture.

This sleep posture monitoring system is largely based on the Raspberry Pi 4 Model B microprocessor combined with a high-performance, ultra-high-frequency (UHF) RFID Reader (QBG12X) and its tags; these components were used to construct an Internet of Things system with integrated artificial intelligence (AI) for sleeping posture recognition and monitoring, as depicted in Figure 1 [23]. Firebase mainly provides cloud services for mobile application devices to replace some or all backend functions (Backend as a Service, BaaS). It also supports Java Script and C++, which can be used in Web and desktop applications, allowing developers to develop applications more efficiently. A system with a peak recognition speed greater than 100/s, can be used to identify and monitor the sleeping postures of individuals in a ward or at home, fully meeting the experimental requirements. The RFID reader connects and communicates with the Raspberry Pi through the WIFI antenna. The IP and port address of the reader are set up via the monitoring interface of the Raspberry Pi. The RFID reader transmits the received data to the Raspberry Pi 4B through the Transmission Control Protocol network. The processed data are inputted to the trained model as features that can be used to recognize the user’s current sleeping posture. Finally, the Raspberry Pi displays the interpreted sleeping posture on the monitoring screen and uploads it to the database via the Internet. The current posture is simultaneously displayed on the graphical user interface, with the time recorded to produce a trend graph for the doctor or user’s reference. The construction of this system provides an accurate sleep recording method without recognizing foreign bodies to improve the user’s sleep experience and to enhance the efficiency of the medical staff.

The experimental sample data were obtained by monitoring the sleeping position of a single person on a bed with RFID tags. The experimental bed at the bottom left in Figure 1 is 200 cm long and 100 cm wide. RFID tags are evenly pasted on the surface of the bed sheet in a 5 × 5 matrix manner and at the same interval. The top tag is placed at the shoulder position just below the neck, and the lowest tag is placed at the calf position above the ankle. Each row of tags is about 30 cm apart, and each column is about 11.4 cm apart. The tags used in the experiment have a frequency range of 860~960 MHz, are 74 mm × 24 mm in size, and are suitable for paper and cloth surfaces. Since the RFID reader has a maximum receiving distance of 5 m, in order to make the signal stable, the RFID reader is placed about 1 m at the foot of the bed, and the transmission intensity is set to 30 dbm. The received signal strength indicator (RSSI) range of the UHF RFID reader is 30~90 and can be converted to −100~−40 dbm. The other reader transmission specifications include 0–33 dbm and an adjustable, maximum reading distance of 5 m as well as a radio frequency range of 902–928 MHz. The reader will decrease the sampling rate as the number of sampled tags decreases, so the sampling rate under 25 tags is 2–3 times in 1 s.

A simple neural network was also previously employed for sleep posture classification [7,12]. However, neural network models are prone to under fitting if inadequately structured or to overfitting the training dataset if structured to meet every single item in the dataset [15]. Deep neural networks (DNNs) also have some disadvantages—they require large amounts of training data, numerous parameters, and careful parameter tuning. As a result, DNN is not suitable for a system that are constrained by a small dataset and low computation and memory costs.

The SVM method was combined with either the quadratic kernel to recognize different sleep postures [16] or the radial basis function kernel to recognize four typical sleep postures [17]. SVMs can also be combined with kurtosis and skewness estimation and principal component analysis (PCA) for sleeping posture classification [18]. Variants of the support vector machine (SVM)-enabled radial basis function (RBF) and SVM-Linear for remote image sensing are proposed and implemented [19]. The results of the proposed system were compared to those obtained by the traditional algorithms (maximum likelihood classifier and minimum distance classifier) and the current state-of-the-art algorithms.

The RFC algorithm has the following advantages [15]: (1) it can handle high-dimensional data (with many features) and does not require feature selection; (2) after training, it can identify the most vital features; (3) it facilitates simple parallel processing at a relatively fast speed; (4) it can be visually displayed for analysis; and (5) it can tolerate large noise and balance errors. Many RFC based applications have been proposed. RFC was initially employed to predict relative humidity in the smart factory environment and showed 82.49% accuracy, which is considered excellent [21]. The random forest classification, decision tree classification, gradient boosting classification, and Naive Bayes classification image processing technologies were used to classify the types of rice leaf disease in Thailand. The best results were obtained by the random forest algorithm, which achieved 69.44% accuracy [22]. Various heart rate variability metrics can be used for driver sleepiness classification. Subjective ratings based on the Karolinska sleepiness scale were used as a ground truth during classifier training [24]. A random forest classifier (RFC) was applied to a dataset with features that contained URL metadata to prevent users from inadvertently accessing phishing websites [25]. The classification performance was compared to those of the linear and nonlinear SVM algorithms when using two datasets. A non-neural-network-style deep model with a lightweight multilayer RFC algorithm [26] consisting of layer-by-layer RFs was proposed for conducting facial expression recognition and for overcoming the disadvantages of the DNN. The RFC method was proposed to combine linear interpolation, matrix combination, and matrix transposition to solve filling problems in large amounts of missing electric power data [27].

In the recursive process of building the classification tree, the classification and regression tree (CART) consistently selects the feature with the smallest Gini information gain in the current dataset as the node partition decision tree. The dichotomy of the CART algorithm can simplify the scale of the decision tree and improve the efficiency at which the trees can be created. To avoid overfitting, the CART decision tree must be pruned.

The operational process of RF classification can be described as follows [17]:

(1) RF first uses bootstrap sampling to create the training data. If *N* samples are presented, each sample has *M* variables (features), and *n* training data are created through random sampling with replacement.

(2) A different random vector *θ_i_* is then generated for each training datum through the random selection of *m* (*m* < *M*) variables and by attempting to split each variable. When the minimum Gini coefficient is reached, the tree is split to create the CART.

(3) The resultant tree grows without pruning.

(4) The results of *n* trees are combined, and the majority voting method is used to cast the predicted value.

The Gini coefficient *I_G_*(*t*) is expressed in the following equation:

(1) $$I_{G}\left(t\right)=\overset{M}{\underset{i=1}{\sum }}p\left(i|t\right)\left(1-p\left(i|t\right)\right)=1-\overset{M}{\underset{i=1}{\sum }}{\left(p\left(i|t\right)\right)}^{2}$$

where p(i|t) represents the proportion of the category *i* at a certain node *t*. If the variables have multiple collinearity or unbalanced data, the RF algorithm can be used [13].

A common strategy used to verify whether the classifier can accurately predict unknown data (testing data) is to separate the dataset into two parts, one of which is considered unknown. The prediction accuracy obtained from the unknown set precisely reflects the classification performance on an independent dataset. An improved version of this procedure is called *K*-fold cross-validation, where the training set is first divided into *K* subsets of equal size, and then one subset is sequentially tested using the classifier trained on the remaining *K* − 1 subsets. Thus, each instance of the whole training set is predicted once, and the cross-validation accuracy is the percentage of data that are correctly classified. In addition, the grid-search method is recommended for the cross-validation procedure to prevent overfitting. The grid search screens all candidate parameter selections and tests every possibility until the best-performing parameter is identified. The principle is similar to that of determining the maximum value in an array.

## 3. Experimental Results

The Raspberry Pi 4 Model B processor [28] consists of a 1.5 GHz Broadcom BCM2711 (quad-core Cortex-A72), upgraded Bluetooth 5.0, two universal serial bus (USB) 2.0 interfaces, two USB 3.0 interfaces, a power supply with a newer USB-C interface, and an onboard memory capacity that is up to 4 GB, which is four times higher than that of the Raspberry Pi 3b. QBG12X is a high-performance UHF RFID all-in-one reader that uses a high-performance INDY R2000 chip solution, efficient signal processing algorithm, excellent multitag anticollision function, and working frequency of 902–928 MHz or 865–868 MHz to provide a high electronic tag reading rate and rapid reading and writing capabilities. It is widely used in various RFID application systems such as in intelligent remote parking lots, personnel access control, logistical storage, production process control, quality traceability, and anticounterfeiting.

The RFID principle for sleeping posture monitoring is that RFID electromagnetic waves cannot penetrate human tissues, so the tags covered by human tissues will not be read by the reader. There are 5 × 5 tags that are evenly set up on the bed sheet, so each posture has one received signal strength indicator (RSSI) value corresponding to a set of 25 tags. The sleeping posture is labeled corresponding to each set of data as follows: supine posture as 1, lying right as 2, lying left as 3, and empty bed status as 0. This process generates the dataset.

A total of 41 participants were enrolled in the experiment, and each participant performed his own version of three sleeping postures (120 sample data points) with slight posture changes in each sleeping position. The participants were 20 to 30 years old, weighed 40 to 110 kg, were between 150 and 185 cm in height, and were healthy. Deleting some duplicate data to ensure the diversity of the information, 3854 samples were obtained from the total of 4920. At the end of the experimental stage, the 3854 samples will be divided, 70% from 29 participants being used for training and the remaining 30% from the other 12 participants being used for testing.

**Figure 2:**
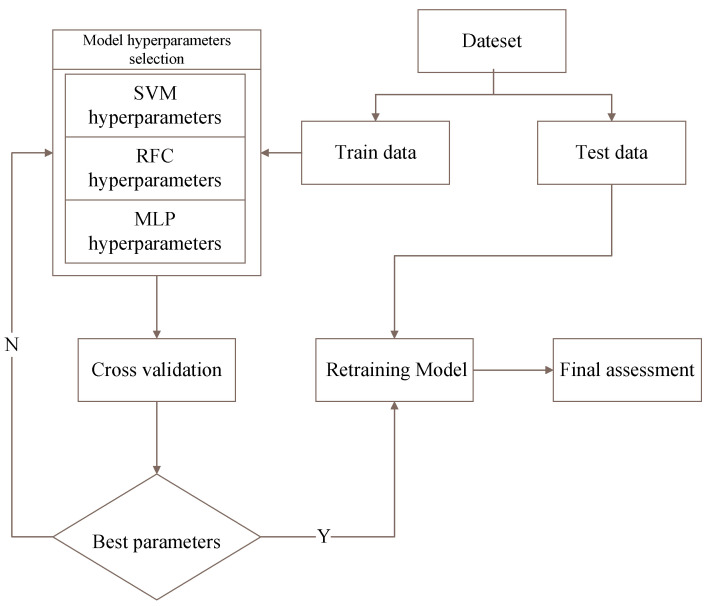
Schematic of the overall parameter selection and model training process.

The proposed RFC-based sleep posture monitoring system was verified using the aforementioned hardware system and datasets. In addition, the experimental results based on RFC were compared to those obtained using the SVM method and MLP algorithm to demonstrate the superior performance of the proposed system. The Scikit-learn function library [29], a free software machine-learning library for the Python programming language, was employed to provide various classification and regression and clustering algorithms including SVMs, RFs, gradient boosting, k-means, and the density-based spatial clustering of applications with noise. The grid search, cross-validation, and evaluation model used in the library were the GridSearchCV sklearn.model_selection, cross_val_score from sklearn.cross_validation, and classification_report from sklearn.metrics, respectively. A schematic of the overall parameter selection and model training process are presented in Figure 2.

For RFC, four parameters were adjusted: the number of subtrees *N* that had the greatest effect on the classifier; the number of maximum features *M* that can complicate or simplify the model; the minimum number of samples for the branch nodes of the tree; and the maximum depth of the decision tree, which can be generally adjusted according to the size of the sample data. The optimized parameter set obtained through the grid search was (177, 1, 20, 9). For the MLP, three parameters were adjusted: the size of the hidden layer and the crucial hyperparameter of the MLP that represents the number of layers and number of neurons in the hidden layer; the number of maximum iterations of the classifier; and the solver function used for weight optimization, which includes the limited-memory Broyden–Fletcher–Goldfarb–Shanno (LBFGS) algorithm, stochastic gradient decent, and adaptive moment estimation. The BFGS algorithm is an iterative method that can be used to solve unconstrained nonlinear optimization problems and that determines the descent direction by preconditioning the gradient with curvature information. The computational complexity of the update of the BFGS matrix without requiring matrix inversion is only $O(n^{2})$ compared to $O(n^{3})$ when using Newton’s method. The optimized parameter set obtained through grid search was ((100, 50), 250, LBFGS). For the SVM, we adjusted three parameters: the penalty coefficient C of the objective function, which is used to balance the classification interval and misclassified samples; one of four kernel functions, namely the radial basis function (RBF), linear, polynomial, and sigmoid functions; and gamma, the coefficient of the RBF kernel function that determines the number of support vectors and affects the generalization performance. The Gaussian RBF kernel is expressed as

(2) $$k(x_{i},x_{j})=\exp(-\gamma {\left\|x_{i}-x_{j}\right\|}^{2}),\;\gamma >0$$

and the optimization problem is

(3) $$\underset{w,b,\xi }{\min}\frac{1}{2}w^{T}w+C\sum_{i=1}^{l}\xi_{i}$$

which is subject to

(4) $$y_{i}(w^{T}\varphi (x_{i})+b)\geq 1-\xi_{i},\;\xi_{i}\geq 0$$

where $(x_{i},y_{i}),\;i=1,\cdots ,l$ is a training set of instance-label pairs, $x_{i}\in R^{n}$ and $y\in {\left\{1,-1\right\}}^{l}$. The optimized parameter set obtained through the grid search was (100, RBF, 1).

Ten *K*-fold cross-validation on the training samples was performed, and the recognition accuracy of the three classifiers was compared (Table 1). This experiment explored the generalization of each model, and the three pretrained models were used to predict the test data. Their prediction accuracy rates as determined by MLP, SVM, and RFC are detailed in Table 2.

**Figure 3:**
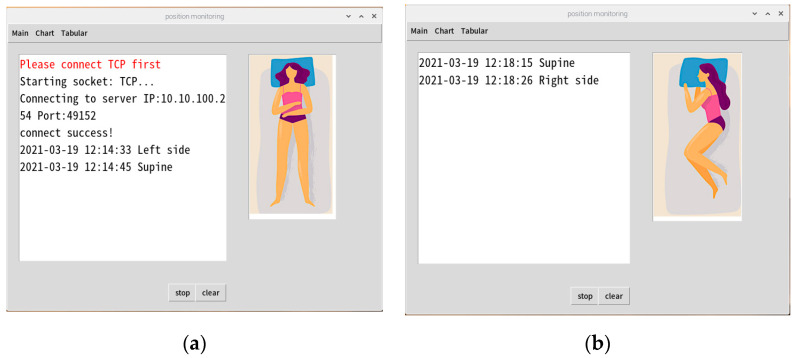

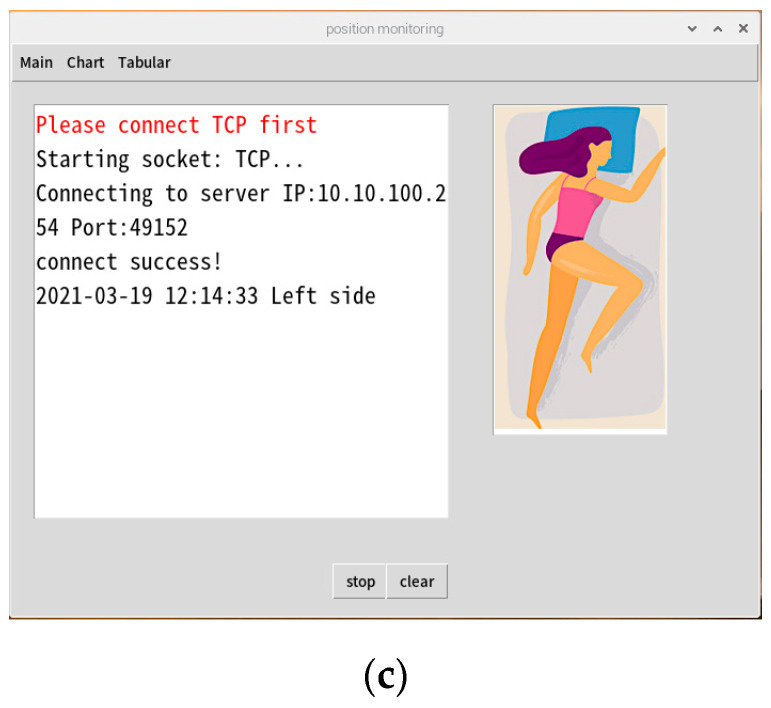
Graphical user interface (GUI) results of the Raspberry Pi for (**a**) a supine position, (**b**) recumbent right, and (**c**) recumbent left.

**Figure 4:**
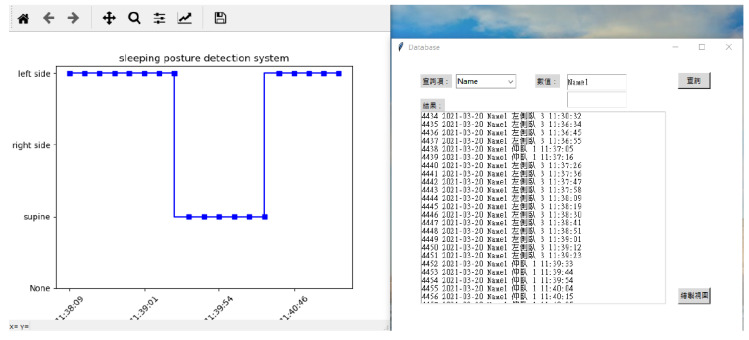
Terminal manager interface and GUI results of the PC terminal.

**Figure 5:**
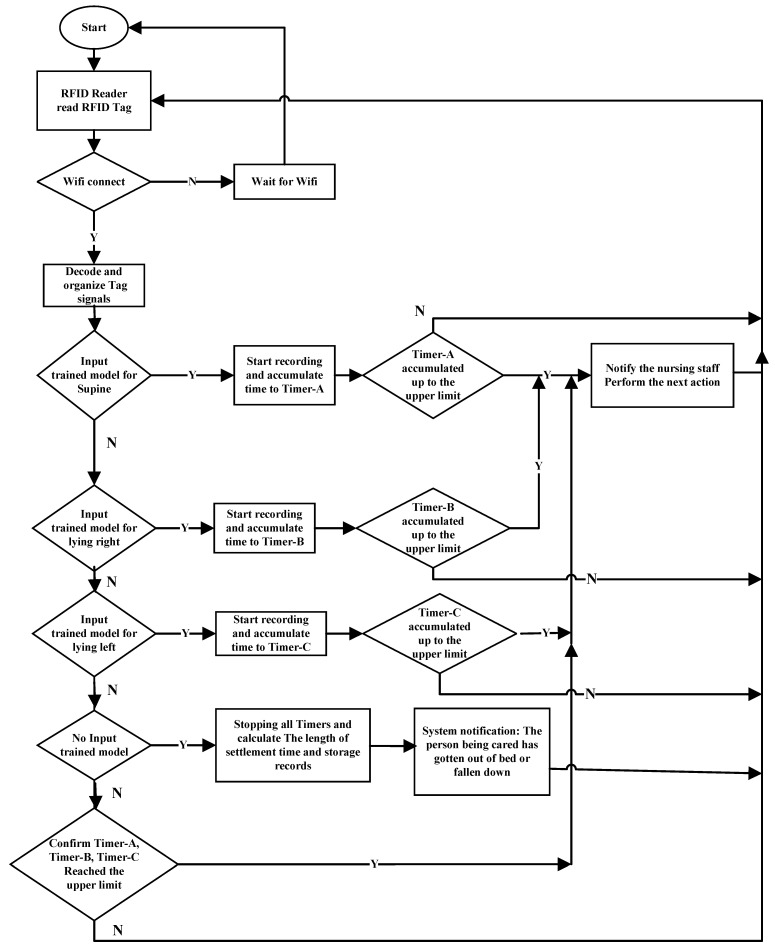
Flowchart of the proposed system.

This system has two interfaces for monitoring the user’s sleeping posture. The first is the Raspberry Pi terminal, which constitutes the monitoring interface of the user terminal. This interface displays the current user’s sleeping posture and a trend graph. Before system initialization, the Internet Protocol, port address of the RFID Reader, sheet tag number, sleeping posture reminder time, and user’s number or name must be inputted. The second interface is the terminal interface for managers or terminal operators; it displays the real-time sleeping posture changes of the user, as depicted in Figure 3. This interface combines the data from all of the clients to form a monitoring management interface that displays the status of the subordinate clients and queries historical data and trend graphs using the local MySQL database, as illustrated in Figure 4. The summary flowchart is presented in Figure 5.

## 4. Discussion

**Figure 6:**
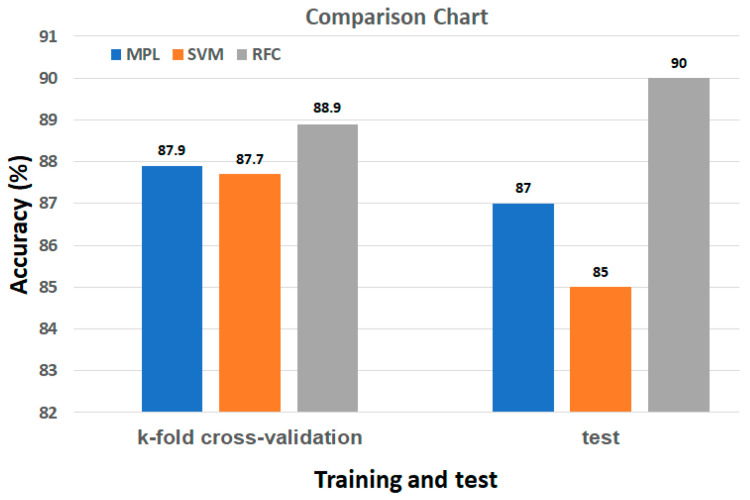
Comparison chart of *K*-fold cross-validation results and prediction of test data.

From Table 1 and Table 2, the comparison chart of the *K*-fold cross-validation results and predictions of the test data is shown in Figure 6. The accuracy rates were largely consistent with the cross-validation results; for the generalization performance and the accuracy of the model for each posture, the RFC exhibited the best performance, with an accuracy of 88.9%. Furthermore, no overfitting occurred.

In terms of privacy protection, with the exception of using RFID to replace a depth camera to capture three-dimensional images of the monitored person, the implementation of the proposed system is such that the sleeping posture results identified by the AI algorithm are sent to the cloud database by Raspberry Pi via MQTT (encrypted process). Users who query the real-time sleeping posture and sleeping posture history data in the cloud database are verified by the system for anti-counterfeiting purposes at login. In addition, all of the monitored objects to be checked are handled in accordance with the relevant regulations of the country in terms of the disclosure of medical information. The user at the front end of the system is only able to know the tag numbers of the RFID. Only the authorized back-end cloud manager will know the person who uses the tags, that is, the relation between tag numbers of the RFID and bed number.

Comparing three RFID-based sleep monitor systems [5,13] and the proposed system, it is easy to see that [13] provided the highest accuracy of 96.7% when using PC, 480 RFID tags, and hierarchical recognition that included image processing and polynomial fitting; Ref. [5] also introduced a PC-based system with an additional 64 RFID tags and used 5-layer CNN to achieve accuracy of 86.284%; however, our proposed system was Raspberry-based and only had 25 RFID tags and used RFC to achieve an accuracy of 88.9%. That is, the proposed AIoT system not only had the characteristics of being low cost and compact, safe, and non-contact sensing, but it also provided accurate and privacy-protected monitoring.

## 5. Conclusions

The proposed system incorporates the Raspberry Pi 4 and RFID Reader and its tags to construct an Internet of Things system with integrated RFC for sleeping posture recognition and monitoring and that avoids sensing foreign bodies and protects user privacy. The experimental results demonstrated that the proposed RFC-based system exhibited the best performance compared with those using SVM and MLP, achieving an accuracy of 88.9%. In a hospital or nursing home scenario, when a patient maintains a sleeping position for a prolonged period of time, the proposed system notifies the nurses are that they should turn the patient over.

With the technological innovation of RFID, the accuracy of this system can be increasingly improved to provide more functions, such as those measuring user temperature and the humidity and moisture in the bed. These tags can also be integrated into clothing to monitor the movement and speed of the wearer in the room. In addition, our future work will determine advanced modifications that can be made to algorithm, and state-of-the-art algorithms will be adopted for the designed system to improve its performance.

## Figures and Tables

**Table 1:** Ten *K*-fold cross-validation results of each classifier.

Classifier	*K*-Fold 1	*K*-Fold 2	*K*-Fold 3	*K*-Fold 4	*K*-Fold 5	*K*-Fold 6	*K*-Fold 7	*K*-Fold 8	*K*-Fold 9	*K*-Fold 10	Average
SVM	92.8%	91.2%	95.6%	82.4%	90.2%	70.4%	87.5%	87.0%	90.6%	91.6%	87.9%
MLP	94.1%	86.8%	95.3%	83.9%	91.2%	75.8%	89.1%	84.6%	90.3%	85.3%	87.7%
RFC	95.3%	97.2%	96.1%	79.8%	85.0%	78.2%	88.6%	86.5%	97.7%	84.6%	88.9%

**Table 2:** Results obtained using the MPL, SVM, and RFC to predict new data.

	Precision (MPL, SVM, RFC)	Recall (MPL, SVM, RFC)	F1-Score (MPL, SVM, RFC)
Supine	82%, 74%, 84%	83%, 90%, 88%	82%, 81%, 86%
Lying right	95%, 99%, 99%	96%, 90%, 93%	95%, 95%, 96%
Lying left	84%, 86%, 86%	82%,74%, 88%	83%, 79%, 87%
Macro avg	87%, 86%, 90%	87%, 85%, 90%	87%, 85%, 90%
weighted avg	87%, 87%, 90%	87%, 85%, 90%	87%, 86%, 90%
Accuracy			87%, 85%, 90%

## Data Availability

Not applicable.
